# Differences in the On- and Off-Tumor Microbiota between Right- and Left-Sided Colorectal Cancer

**DOI:** 10.3390/microorganisms9051108

**Published:** 2021-05-20

**Authors:** Oliver Phipps, Mohammed N. Quraishi, Edward A. Dickson, Helen Steed, Aditi Kumar, Austin G. Acheson, Andrew D. Beggs, Matthew J. Brookes, Hafid Omar Al-Hassi

**Affiliations:** 1 Research Institute in Healthcare Science, Faculty of Science and Engineering, University of Wolverhampton, Wolverhampton WV1 1LY, UK; o.phipps@wlv.ac.uk (O.P.); helen.steed@nhs.net (H.S.); aditikumar@nhs.net (A.K.); matthew.brookes@nhs.net (M.J.B.); 2 Surgical Research Laboratory, Institute of Cancer & Genomic Science, University of Birmingham, Birmingham B15 2TQ, UK; M.N.Quraishi@bham.ac.uk (M.N.Q.); a.beggs@bham.ac.uk (A.D.B.); 3 Microbiome Treatment Centre, University of Birmingham, Birmingham B15 2TT, UK; 4 Department of Gastroenterology, University Hospitals Birmingham NHS Foundation Trust, Birmingham B15 2TH, UK; 5 NIHR Biomedical Research Centre in Gastrointestinal and Liver Diseases, Nottingham University Hospitals NHS Trust, Nottingham NG7 2UH, UK; edward.dickson1@nottingham.ac.uk (E.A.D.); austin.acheson@nottingham.ac.uk (A.G.A.); 6 Department of Colorectal Surgery, Nottingham University Hospitals NHS Trust, Nottingham NG5 1PB, UK; 7 Department of Gastroenterology, Royal Wolverhampton Hospitals NHS Trust, Wolverhampton WV10 0QP, UK

**Keywords:** right- and left-sided colorectal cancer, gut microbiome, 16S rRNA, tumor microbiota, proximal and distal colon

## Abstract

This study aims to determine differences in the on- and off-tumor microbiota between patients with right- and left-sided colorectal cancer. Microbiome profiling of tumor and tumor-adjacent biopsies from patients with right-sided (*n* = 17) and left-sided (*n* = 7) colorectal adenocarcinoma was performed using 16S ribosomal RNA sequencing. Off-tumor alpha and beta diversity were significantly different between right- and left-sided colorectal cancer patients. However, no differences in on-tumor diversity were observed between tumor locations. Comparing the off-tumor microbiota showed the right colon to be enriched with species of the Lachnoclostridium, Selenomonas, and Ruminococcus genera. Whereas the left colon is enriched with Epsilonbacteraeota phylum, Campylobacteria class, and Pasteurellales and Campylobacterales orders, in contrast, the on-tumor microbiota showed relatively fewer differences in bacterial taxonomy between tumor sites, with left tumors being enriched with Methylophilaceae and Vadin BE97 families and Alloprevotella, Intestinibacter, Romboutsia, and Ruminococcus 2 genera. Patients with left-sided colorectal cancer had large taxonomic differences between their paired on- and off-tumor microbiota, while patients with right-sided colorectal cancer showed relatively fewer taxonomic differences. Collectively, this suggests that the right and left colon show distinctive bacterial populations; however, the presence of a colonic tumor leads to a more consistent microbiota between locations.

## 1. Introduction

**Figure 1:**
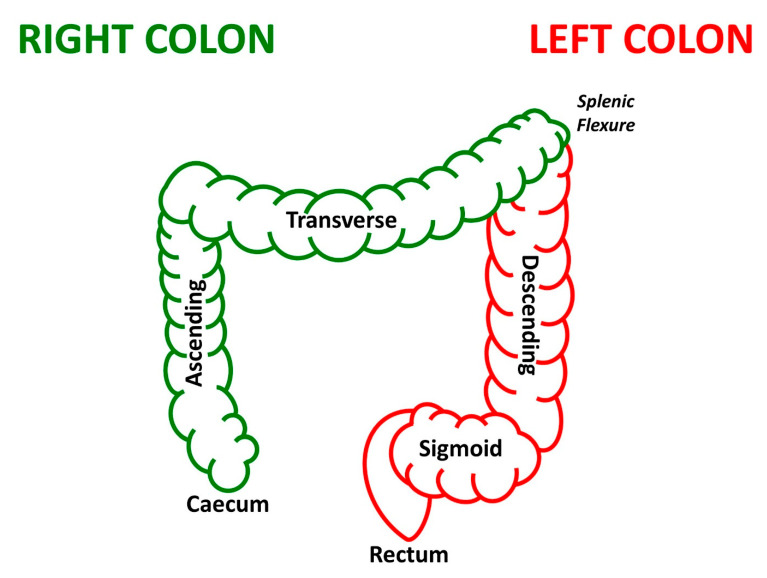
Schematic diagram of the right and left colon in relation to the colonic splenic flexure. The right colon (green) consists of the caecum, ascending, and transverse colon and the left colon (red) consists of the descending colon, sigmoid colon, and rectum.

The term colorectal cancer encompasses a heterogeneous group of tumors of the lower gastrointestinal tract. Cancer location can be defined in relation to the colonic splenic flexure (Figure 1), with tumors of the caecum, ascending and transverse colon, termed right-sided colorectal cancer, and tumors of the descending colon, sigmoid colon, and the rectum, termed left-sided colorectal cancer [1,2]. The right and left colon have distinctive embryological origins, developing from the midgut and hindgut, respectively [3]. These two anatomical colonic locations are supplied by different blood supplies, with the right colon being supplied by the superior mesenteric artery and the left colon being supplied by the inferior mesenteric artery [3,4].

Disparities between the right and left colon translate into observable differences in molecular and clinical characteristics between tumors in these locations. These include right-sided tumors more commonly associated with microsatellite instability, being highly immunogenic, and presenting with BRAF mutations. Whereas, left-sided tumors present with chromosomal instability, often showing mutations in *APC*, *P53*, and *SMAD4*, and are associated with amplified epidermal growth factor receptor signaling [5]. These differences may be accountable for the variability in cancer incidence and prognosis between tumor locations. With right-sided tumor patients tending to have a lower incidence with more advanced tumors and worse prognosis, compared to the left-sided tumor. Furthermore, right-sided tumors are more common in older females, whereas left-sided tumors are more frequent in younger males [1,5]. With right- and left-sided colorectal tumors presenting with such differences, further understanding the biological mechanisms underpinning cancer initiation and progression, and how these differ between the right and left colon, can aid in disease prevention and treatment.

A multistep pathogenesis is involved in the etiology of sporadic colorectal tumors, with genetics, lifestyle, and environment all known to contribute to colorectal carcinogenesis [6]. Along with this, the gut bacterial microbiota and their metabolites have also been shown to both promote and protect against colorectal cancer [7]. Therefore, identifying the bacterial populations in the tumor-associated (on-tumor) and tumor-adjacent (off-tumor) microbiota, and how they differ between the right and left colon, has the potential to be beneficial in predicting disease outcomes and aid in patient stratification [8,9].

Previous studies have attempted to determine differences in the gut microbiota between right- and left-sided colorectal cancer [10,11]. Flemer et al. (2017) showed that there was no observable difference between the on- and off-tumor microbiota, along with showing that the on-tumor microbiota from the right and left colon were significantly different [10]. However, as subsequently discussed by Al-Hassi et al. (2018), patients with right-sided colorectal tumors more commonly develop iron deficiency anemia relative to those with left-sided tumors, hence more often requiring iron therapy [12]. Enteral iron supplementation, often given to treat anemia, has been shown to alter the colonic microbiota, potentially through increasing gut luminal iron availability for bacterial cell proliferation [13]. The occurrence of iron deficiency anemia and the use of enteral iron supplementation was not assessed by the authors in the Flemer study and has the possibility to be accountable for differences observed [10]. Therefore, when assessing the gut microbiota in colorectal cancer, ensuring consistency in the prevalence of iron deficiency anemia and iron therapy is essential [14,15].

To our knowledge, this pilot study is the first to assess both the tumor-associated and tumor-adjacent microbiota between right- and left-sided colorectal cancer patients, while ensuring consistency in the prevalence of iron deficiency anemia and iron therapy. All colorectal cancer patients included in this study had iron deficiency anemia and received parenteral iron therapy prior to surgery. Parenteral iron therapy was given in order to limit the influence of increasing gut iron concentration on the gut microbiota, associated with enteral iron therapy [13,16].

## 2. Materials and Methods

### 2.1. Study Population and Sample Collection

Twenty-four anemic patients with non-metastatic histologically proven colorectal adenocarcinoma, presenting with right-sided (*n* = 17) and left-sided (*n* = 7) tumors, from the intravenous iron in colorectal cancer associated anemia (IVICA) trial, were included in the study. All patients selected for this study received intravenous iron (ferric carboxymaltose—Ferinject ™; Vifor Pharma, Glattbrugg, Switzerland) prior to surgery, dosed by weight and hemoglobin in accordance with the summary of product characteristics [17]. Treatment was administered at least 14 days preoperatively, with any pre-existing oral iron supplements being discontinued at this point. Anemia was defined as having a hemoglobin level at least 10 g/L below the sex-specific World Health Organization definition (women ≤ 120 g/L, men ≤ 130 g/L). Patients were excluded from the study if they were currently receiving chemotherapeutic treatment, had anemia prior to colorectal cancer diagnosis, or pre-existing hematological disease. Detailed patient demographics can be found in Table 1. Colorectal tumor biopsies and paired tumor-adjacent colonic mucosal tissue biopsies were obtained post-surgery.

### 2.2. DNA Extraction and 16S Ribosomal RNA Amplicon Sequencing

Bacterial DNA was obtained using a modified protocol of Qiagen All Prep DNA/RNA Mini Kit (Qiagen, Hilden, Germany). Colorectal tumor and paired tumor-adjacent biopsies were mechanically lysed using 5 mm steel bead (Qiagen) and 0.1 mm Zirconia/Silica beads (Strateck, Suffolk, UK) with a TissueLyser (Qiagen), followed by enzymatic and heat lysis. Extracted microbial DNA was used for 16S ribosomal RNA (rRNA) gene amplification and sequencing to determine the mucosal-adherent microbiota according to the Earth Microbiome project protocol [18]. Using primers targeted to the V4 region (515F-806R), the 16S rRNA genes were amplified in technical triplicates. This was performed using a single-step, single-indexed polymerase chain reaction (PCR). DNA extraction and 16S rRNA gene PCR were both performed in batch with appropriate multiple-reagent based negative controls. Paired-end sequencing (2 × 250 base pairs) was completed in a single batch using the Illumina MiSeq platform (Illumina, San Diego, CA, USA).

### 2.3. Statistical Analysis

Microbial bioinformatic analysis was achieved using the Quantitative Insight Into Microbial Ecology 2 (QIIME2) pipeline [19]. High-quality reads were clustered into operational taxonomic units (OTUs), reads with a 99% sequence identity were allocated to a single OTU and were assigned bacterial taxonomy using the Silva-132-99% OTU database [20]. Alpha diversity was assessed using the Mann–Whitney U test comparing variation in Abundance-based Coverage Estimator (ACE), Chao1, and observed OTUs between groups. Beta diversity was assessed using permutational multivariate analysis of variance (PERMANOVA) comparing Jaccard similarity coefficients, with distances between groups plotted using principal coordinate analysis (PCoA). PCoA and alpha diversity metrics were mapped using the R package “ggplot2” [21]. Comparisons of relative abundances of taxa between locations and sample types were assessed using a linear discriminant analysis (LDA) effect size (LEfSe). LEfSe uses a non-parametric Kruskal–Wallis rank-sum test to identify taxa with significantly different normalized relative abundances and performs an LDA to determine an effect size of each taxon. Taxa with an LDA greater than 2 with a *p*-value ≤ 0.05 were considered significant [22].

## 3. Results

### 3.1. On- and Off-Tumor Bacterial Diversity between the Right and Left Colon

**Figure 2:**
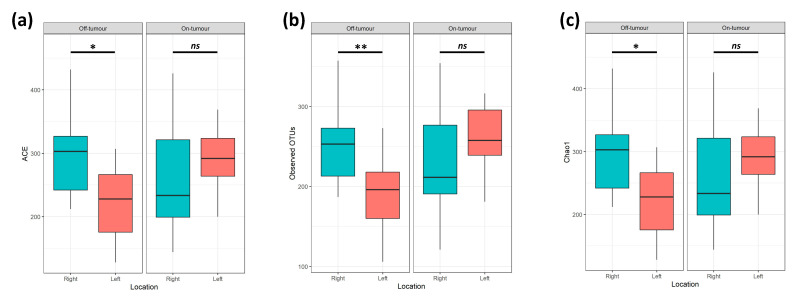
On- and off-tumor alpha diversity between right and left colon. Alpha diversity metrics (**a**) Abundance-based coverage estimate (ACE), (**b**) Observed operational taxonomic units (OTUs), and (**c**) Chao1 were significantly greater in the right compared to the left off-tumor microbiota. On-tumor alpha diversity metrics showed no significant differences between tumor locations. (* *p* ≤ 0.05, ** *p* ≤ 0.01, *ns p* > 0.05).

**Figure 3:**
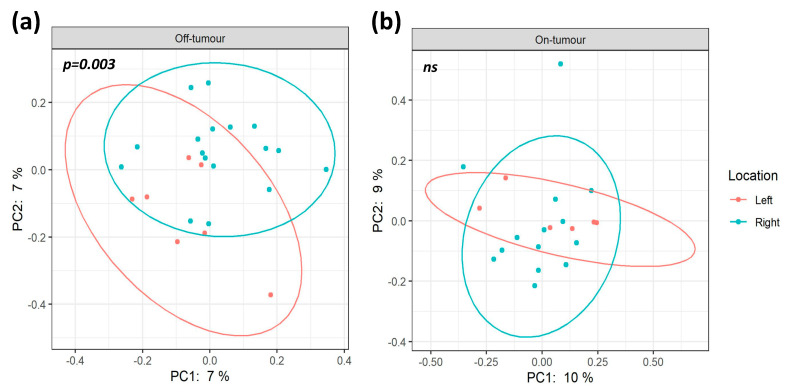
On- and off-tumor beta diversity between right and left colon. Principle coordinate analysis (PCoA) plots based on Jaccard distances show significantly distinct bacterial community clusters (*p* = 0.003) between the off-tumor microbiota from the right and left colon (**a**). On-tumor microbiota from the right and left colon show no significant differences (*ns*) *(***b**).

A total of 4.4 million reads (an average of 109,122 reads/sample) were obtained following quality control, a sampling depth of 8000 reads/sample was chosen following rarefaction. Comparison of alpha diversity metrics shows that the off-tumor microbiota of patients with right-sided colorectal cancer showed significantly greater ACE (Bacterial Abundance), Observed OTUs (Species Richness), and Chao1 (Bacterial Diversity), compared to left-sided colorectal cancer patients (Figure 2; *p* < 0.05). Consistent with this, the Jaccard similarity assessed beta diversity, showing that the off-tumor microbiota of right- and left-sided colorectal cancer patients formed significantly distinct bacterial community clusters (Figure 3a; *p* = 0.003). In contrast, the on-tumor microbiota showed no differences in alpha diversity between right- and left-sided colorectal cancer patients (Figure 2; *ns*). Furthermore, no significant differences in beta diversity were observed between right- and left-sided colorectal tumors (Figure 3b; *ns*). Collectively this suggests that bacterial diversity is greater in the right colon compared to the left; however, the presence of a colonic tumor leads to a more consistent bacterial diversity between locations.

### 3.2. Gut Phylogenetic Profiles between Right- and Left-Sided Colorectal Cancer Patients

**Figure 4:**
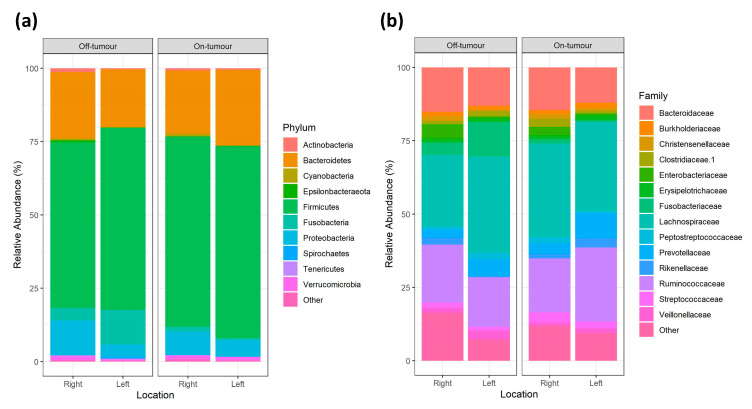
On- and off-tumor phylogenetic profiles of gut bacterial populations in the right and left colon. Relative abundance of on- and off-tumor mucosal adherent gut bacterial microbiota at phylum (**a**) and family (**b**) taxonomic levels in right and left colon.

Firmicutes, Bacteroides, Proteobacteria, and Fusobacteria constitute >95% of bacteria phyla in each group, showing largely consistent phylum relative abundance between locations (Figure 4a). The 3 most dominant bacterial families across all locations were *Lachnospiraceae*, *Ruminococcaceae*, and *Bacteroidaceae*. However, there is variation in the subsequent most abundant families between locations. Within the off-tumor microbiota, the next most abundant family was *Fusobacteriaceae*, followed by *Enterobacteriaceae* in the right colon and *Prevotellaceae* in the left colon. While in the on-tumor microbiota the next most abundant family is *Prevotellaceae*, followed by *Streptococcaceae* in the right-sided tumor and *Rikenellaceae* in the left-sided tumors (Figure 4b).

### 3.3. Comparison of Bacterial Taxa between the Right and Left Colon

**Figure 5:**
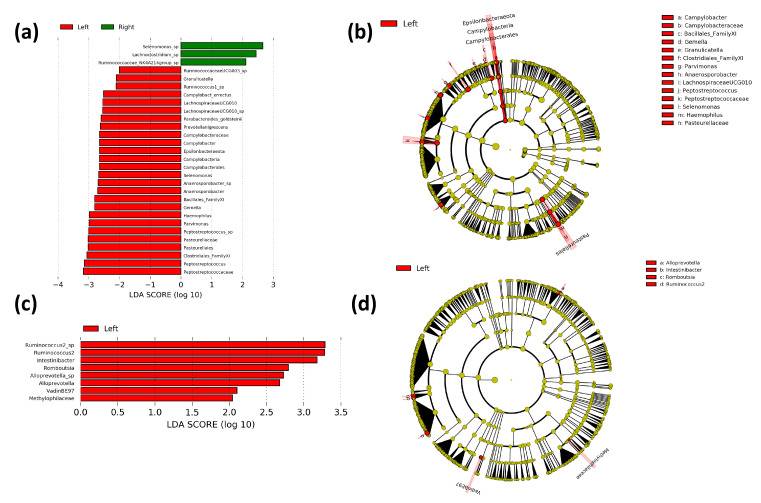
Linear discriminant analysis (LDA) effect size (LEfSe) comparing right and left bacterial taxa in the on- and off-tumor microbiota. Histograms of LDA scores for differentially abundant bacterial taxa between right and left colon in off-tumor (**a**) and on-tumor (**c**). LEfSe cladogram representing differentially abundant bacterial groups in off-tumor (**b**) and on-tumor (**d**) microbiota between right and left colon. Differentially abundant taxa at the genus taxonomic levels or higher were included. Taxa and nodes highlighted in green were more significant in the right colon and red in the left colon.

Differences in gut bacterial populations between right- and left-sided colorectal cancer patients were assessed using LEfSe to determine bacterial taxa that are significantly enriched between locations. The off-tumor microbiota of right-sided colorectal cancer patients showed greater abundances of species of the *Lachnoclostridium*, *Selenomonas*, and *Ruminococcus* genera. Whereas, the off-tumor microbiota of left-sided colorectal cancer patients is enriched with Epsilonbacteraeota phylum, Campylobacteria class, Pasteurellales and Campylobacterales orders, *Campylobacteraceae*, *Bacillales Family XI*, *Clostridiales Family XI*, *Peptostreptococcaceae* and *Pasteurellaceae* families, and *Campylobacter*, *Gemella*, *Granulicatella*, *Parvimonas*, *Anaerosporobacter*, *Lachnospiraceae* (UCG010), *Peptostreptococcus*, *Selenomonas,* and *Haemophilus* genera (Figure 5a,b). The on-tumor microbiota of left-sided cancer patients showed greater abundances of *Methylophilaceae* and *Vadin BE97* families and *Alloprevotella*, *Intestinibacter*, *Romboutsia,* and *Ruminococcus 2* genera, compared to right-sided colorectal cancer patients (Figure 5c,d). Collectively, these results support the diversity data, with the off-tumor microbiota showing large differences in bacterial populations between the right and left colon. In contrast, the on-tumor microbiota seems less affected by location, supporting a cancer-defined microbiota that is more constant between the right and left colon.

### 3.4. Difference in Paired On- and Off-Tumor Bacterial Taxa in Right- and Left-Sided Colorectal Cancer Patients

**Figure 6:**
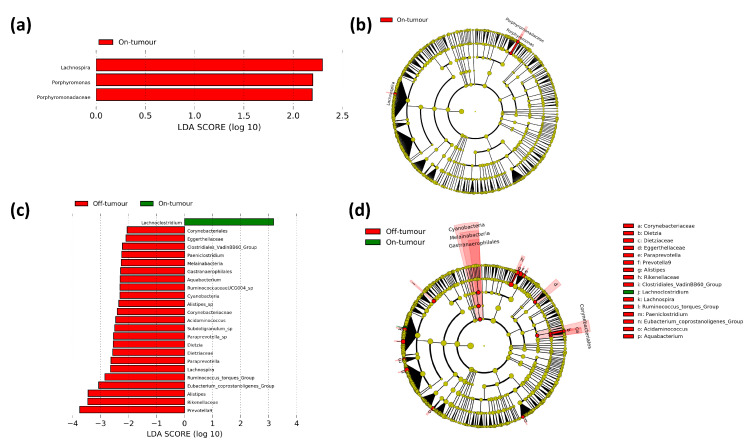
Comparison of paired on- and off-tumor microbiota between right and left colon. LDA scores for differentially abundant bacterial taxa between paired on- and off-tumor microbiota in the right (**a**) and left (**c**) colon. LEfSe cladogram demonstrating differentially abundant bacterial taxa between paired on- and off-tumor microbiota in right (**b**) and left (**d**) colon. Differentially abundant taxa at the genus taxonomic levels or higher were included. Taxa and nodes highlighted in red were more significant in the off-tumor microbiota and green in the on-tumor microbiota.

In order to assess the differences in bacterial taxa between the tumor-associated and tumor-adjacent microbiotas in each location, we performed a LEfSe comparing paired on- and off-tumor bacterial taxa in right- and left-sided colorectal cancer patients. In the left-sided colorectal cancer patients, 24 bacterial taxa were differentially enriched between the on- and off-tumor microbiota. These include the *Lachnoclostridium* genus which was enriched in the on-tumor microbiota and the Cyanobacteria phylum, Melainabacteria class, Gastranaerophilales, and Corynebacteriales orders, *Dietziaceae*, *Corynebacteriaceae*, *Eggerthellaceae*. *Rikenellaceae* and *Clostridiales vadin BB60 group* families, *Dietzia*, *Paraprevotella*, *Prevotella 9*, *Alistipes*, *Lachnospira*, *Ruminococcus torques group*, *Paeniclostridium*, *Eubacterium coprostanoligenes group*, *Acidaminococcus* and *Aquabacterium* genera which were enriched in the off-tumor microbiota (Figure 6c,d). In the right-sided colorectal cancer patients, there were 3 bacterial taxa differentially enriched between the on- and off-tumor microbiota. These include the *Porphyromonadaceae* family, and *Lachnospira* and *Porphyromonas* genera, which were more abundant in the on-tumor compared to the off-tumor microbiota (Figure 6a,b). Collectively, this suggests that patients with right-sided colorectal cancer have an on- and off-tumor microbiota that is relatively consistent, showing only small differences in bacterial taxa lower taxonomic levels. In contrast, patients with left-sided colorectal cancer have an on- and off-tumor microbiota that shows distinct bacterial populations, showing differences at the phylum, class, and order taxonomic levels.

## 4. Discussion

Previous studies have attempted to unravel the complex relationship between gut bacteria and colorectal cancer [9,10]. However, the composition of the colonic microbiota can be influenced by a multitude of variables, such as medication. Hence, this leaves the potential for the study of the gut microbiota in pathological conditions to be confounded by discrepancies in therapeutic interventions [14,15]. To the best of our knowledge, this study is the first time that the colonic on- and off-tumor microbiota has been studied in right- and left-sided colorectal cancer patients with iron deficiency anemia.

This study shows that the on- and off-tumor microbiota between right- and left-sided colorectal cancer patients show differential microbial diversity and bacterial taxa. Off-tumor alpha diversity is significantly greater in right- compared to left-sided colorectal cancer patients, showing greater bacterial diversity, abundance, and richness. Furthermore, off-tumor beta diversity shows significantly different bacterial community clusters between right- and left-sided colorectal cancer patients. Comparison of off-tumor taxonomy shows there to be 29 bacterial taxa that are differentially enriched between the right and left colon. These differences in off-tumor bacterial populations between the right and left colon are potentially explained by differences in colonic nutrient availability. Nutrient availability is greatest in the proximal colon and decreases towards the distal colon, through nutrients being reabsorbed in the colon and being utilized by residential bacteria [23]. Therefore, this potentially creates differential nutrient niches available for colonic bacteria between the right and left colon, which may explain why the right colon shows greater bacterial diversity compared to the left [24]. Out of the 29 bacterial taxa that were differentially enriched within the off-tumor microbiota, 26 taxa were significantly enriched within the left colon. With the potential differences in nutrient niches between the right and left colon, these bacterial taxa may be specially adapted to thrive within the potentially nutrient-deprived environment within the left colon. Whereas they may be outcompeted by more dominant bacteria that thrive in the potential nutrient-rich environment in the right colon. These more specialized taxa within the left colon may dominate the microbiota, explaining why a lower bacterial diversity may be present. Whereas in the right colon there is a greater number of bacteria which are more evenly spread, contributing to greater bacterial diversity. However, as less of these bacterial taxa dominate, this leads them to not being as significantly enriched when compared to the left colon. In contrast, the on-tumor microbiota shows no differences in alpha and beta diversity between right- and left-sided colorectal cancer patients. Comparison of on-tumor taxonomy shows there to be 8 bacterial taxa that are differentially enriched between the right and left colon. Collectively, this suggests that the on-tumor microbiota between right- and left-sided colorectal cancer patients are relatively more consistent when compared to the differences observed in the off-tumor microbiota, suggesting that the on-tumor microbiota between the right and left colon is not as influenced by colonic nutrient availability, potentially supporting a cancer-defined microbiota that is less affected by tumor location.

Cancer-associated microbiota are influenced by the tumor microenvironment, and this is regulated by many factors, such as the mucosal immune system, genetic and epigenetic factors, and vascular infiltration. How the cancer-associated microbiota develops depends on these factors, all of which are also altered between the right and left colon. Hence, this leaves the potential for alterations between the on- and off-tumor microbiota to be differentially regulated between patients with right- and left-sided colorectal tumors [5,7,11]. Comparison of bacterial taxa between paired on- and off-tumor microbiota showed that patients with right-sided colorectal cancer had a relatively consistent on- and off-tumor microbiota, with only 3 bacterial taxa being differentially enriched. In contrast patients with left-sided colorectal cancer had a more varied on- and off-tumor microbiota, showing 24 differentially enriched bacterial taxa. This supports a previous study by Thomas et al. (2016) comparing the microbiota of rectal (left-sided) cancer patients and healthy controls. The authors showed there to be significant differences in bacterial taxa and diversity between cancerous and healthy rectal microbiota [25]. Collectively this implies that the tumor-defined microbiota may be more consistent to the microbiota found in the right colon compared to the left. Suggesting that there is a shift in the left tumor microbiota away from the typical microbiota found in the left colon, becoming more similar to the right colonic microbiota. Along with patients with right-sided colorectal cancer developing symptoms later, this may also explain why right-sided tumors tend to be more advanced and larger compared to left-sided tumors, as the right colonic microbiota is primed to support colorectal cancer progression [1].

Previous studies comparing the microbiota between the right and left colon, such as those by Flemer et al. (2017) and Gao et al. (2015), have presented differences in the on-tumor microbiota between the proximal and distal colon. These studies have accounted for medicinal intervention with antibiotics prior to surgery; however, neither study assessed the prevalence of iron deficiency anemia or enteral iron therapy in their cohort [10,11]. With iron deficiency anemia tending to be unequally prevalent between right- and left-sided colorectal cancer patients, this may influence the differences observed in the tumor microbiota between locations [26]. As enteral iron therapy has been shown to alter the gut microbiota, this leaves the potential to present with differential bacterial populations compared to patients treated with parenteral iron, or those who are not iron deficient [13,16].

This pilot study provides a novel insight into the on- and off-tumor microbial profiles between right- and left-sided colorectal cancer patients. Suggesting a varied microbiota between the right and left colon; however, the presence of a colonic tumor leads to a more consistent cancer-defined microbiota. This is potentially through the left tumor microbiota being more similar to the microbiota found in the right colon, suggesting that the right colonic microbiota may be more favorable to support colonic tumors. Despite the relatively small sample size of this study, we were able to infer significant differences between right- and left-sided colorectal cancer patients, which can form the foundation for further large-scale explorative studies. Future studies may overcome some limitations of our study by accounting for further confounding variables such as diet. As well as including a more diverse study population, ensuring a more balanced spread of patient characteristics such as gender and tumor stage between groups. Further clinical and translational work is required to understand the influence of iron supplementation on the gut microbiota in right- and left-sided colorectal cancer, how this can influence the stage of disease, and to assess any causal relationship between colonic bacterial populations and oncological outcomes.

## Figures and Tables

**Table 1:** Patient cohort demographics. Categorical variables are presented with percentages. Continuous variables are presented as mean value [standard deviation] or * median value [interquartile range]. Hb, hemoglobin. CR-POSSUM, ColoRectal Physiological and Operative Severity Score for the enumeration of Mortality and morbidity. ASA, American Society of Anesthesiologist.

Patient Characteristics	Right-Sided Colorectal Cancer (*n* = 17)	Left-Sided Colorectal Cancer (*n* = 7)
Age	75.3 [7.5]	74 [7.7]
Male	9 (53%)	7 (100%)
Female	8 (47%)	0 (0%)
Height, m	1.67 [0.08]	1.77 [0.08]
Weight, kg	75.6 [12.8]	88.5 [24.0]
Inclusion Hb, g/L	97.1 [14.1]	102.9 [10.0]
Recruitment ferritin, μg/L *	25 [15–55]	10 [8–49]
Recruitment transferrin saturation, % *	2.6 [2.3–3.6]	2.8 [2.6–3.3]
Duration of iron treatment, days *	21 [15–35]	28 [15–43]
Tumor Stage		
T ≤ 2	1 (6%)	0 (0%)
T3	3 (18%)	5 (71%)
T4	13 (76%)	2 (29%)
Preoperative Risk Assessment		
ASA fitness status classification		
I–II	7 (41%)	2 (29%)
III–IV	10 (59%)	5 (71%)
CR-POSSUM mortality score, % *	3.5 [2.5–9.3]	3.5 [2.6–6.6]

## Data Availability

The datasets generated and analyzed during the study are available from the corresponding author on reasonable request.

## References

[1] Baran B., Ozupek N.M., Tetik N.Y., Acar E., Bekcioglu O., Baskin Y. (2018). Difference Between Left-Sided and Right-Sided Colorectal Cancer: A Focused Review of Literature. Gastroenterol. Res..

[2] Mukund K., Syulyukina N., Ramamoorthy S., Subramaniam S. (2020). Right and left-sided colon cancers—Specificity of molecular mechanisms in tumorigenesis and progression. BMC Cancer.

[3] Kwak H.D., Ju J.K., Lee S.Y., Kim C.H., Kim Y.J., Kim H.R. (2021). Comparison of Right-side and Left-side Colon Cancers Following Laparoscopic Radical Lymphadenectomy. J. Investig. Surg..

[4] Manyama M., Malyango A., Raoof A., Mligiliche N.L., Msuya C., Nassir N., Mtui E. (2019). A variant source of arterial supply to the ascending, transverse and descending colon. Surg. Radiol. Anat..

[5] De Renzi G., Gaballo G., Gazzaniga P., Nicolazzo C. (2020). Molecular Biomarkers according to Primary Tumor Location in Colorectal Cancer: Current Standard and New Insights. Oncology.

[6] Balchen V., Simon K. (2016). Colorectal cancer development and advances in screening. Clin. Interv. Aging.

[7] Alhinai E.A., Walton G.E., Commane D.M. (2019). The Role of the Gut Microbiota in Colorectal Cancer Causation. Int. J. Mol. Sci..

[8] Flemer B., Warren R.D., Barrett M.P., Cisek K., Das A., Jeffery I.B., Hurley E., O’Riordain M., Shanahan F., O’Toole P.W. (2017). The oral microbiota in colorectal cancer is distinctive and predictive. Gut.

[9] Flemer B., Herlihy M., O’Riordain M., Shanahan F., O’Toole P.W. (2018). Tumour-associated and non-tumour-associated microbiota: Addendum. Gut Microbes.

[10] Flemer B., Lynch D.B., Brown J.M.R., Jeffery I.B., Ryan F.J., Claesson M.J., O’Riordain M., Shanahan F., O’Toole P.W. (2017). Tumour-associated and non-tumour-associated microbiota in colorectal cancer. Gut.

[11] Gao Z., Guo B., Gao R., Zhu Q., Qin H. (2015). Microbiota Disbiosis is Associated with Colorectal Cancer. Front. Microbiol..

[12] Al-Hassi H.O., Ng O., Brookes M. (2018). Tumour-associated and non-tumour-associated microbiota in colorectal cancer. Gut.

[13] Phipps O., Al-Hassi H., Quraishi M., Dickson E., Segal J., Steed H., Kumar A., Acheson A., Beggs A., Brookes M. (2021). Oral and Intravenous Iron Therapy Differentially Alter the On- and Off-Tumor Microbiota in Anemic Colorectal Cancer Patients. Cancers.

[14] Phipps O., Al-Hassi H.O., Quraishi M.N., Kumar A., Brookes M.J. (2020). Influence of Iron on the Gut Microbiota in Colorectal Cancer. Nutrients.

[15] Weersma R.K., Zhernakova A., Fu J. (2020). Interaction between drugs and the gut microbiome. Gut.

[16] Lee T., Clavel T., Smirnov K., Schmidt A., Lagkouvardos I., Walker A., Lucio M., Michalke B., Schmitt-Kopplin P., Fedorak R. (2016). Oral versus intravenous iron replacement therapy distinctly alters the gut microbiota and metabolome in patients with IBD. Gut.

[17] Keeler B.D., Simpson J.A., Ng O., Padmanabhan H., Brookes M.J., Acheson A.G., Banerjea A., Walter C., Maxwell-Armstrong C., Williams J. (2017). Randomized clinical trial of preoperative oral versus intravenous iron in anaemic patients with colorectal cancer. J. Br. Surg..

[18] Earth Microbiome Project (2020). Illumina 16s PCR Protocols. https://www.earthmicrobiome.org/protocols-and-standards/16s/.

[19] Bolyen E., Rideout J.R., Dillon M.R., Bokulich N.A., Abnet C.C., Al-Ghalith G.A., Alexander H., Alm E.J., Arumugam M., Asnicar F. (2019). Reproducible, interactive, scalable and extensible microbiome data science using QIIME 2. Nat. Biotechnol..

[20] Quast C., Pruesse E., Yilmaz P., Gerken J., Schweer T., Yarza P., Peplies J., Glöckner F.O. (2013). The SILVA Ribosomal RNA Gene Database Project: Improved Data Processing and Web-Based Tools. Nucleic Acids Res..

[21] Wickham H. (2016). ggplot2: Elegant Graphics for Data Analysis.

[22] Segata N., Izard J., Waldron L., Gevers D., Miropolsky L., Garrett W.S., Huttenhower C. (2011). Metagenomic biomarker discovery and explanation. Genome Biol..

[23] Lyra A., Forssten S., Rolny P., Wettergren Y., Lahtinen S.J., Salli K., Cedgård L., Odin E., Gustavsson B., Ouwehand A.C. (2012). Comparison of Bacterial Quantities in Left and Right Colon Biopsies and Faeces. World J. Gastroenterol. WJG.

[24] Sun J., Kato I. (2016). Gut Microbiota, Inflammation and Colorectal Cancer. Genes Dis..

[25] Thomas A.M., Jesus E.C., Lopes A., Aguiar J.S., Begnami M.D., Rocha R.M., Carpinetti P.A., Camargo A.A., Hoffmann C., Freitas H.C. (2016). Tissue-Associated Bacterial Alterations in Rectal Carcinoma Patients Revealed by 16S rRNA Community Profiling. Front. Cell. Infect. Microbiol..

[26] Acher P.L., Al-Mishlab T., Rahman M., Bates T. (2003). Iron-deficiency anaemia and delay in the diagnosis of colorectal cancer. Color. Dis..

